# CCDC42 Localizes to Manchette, HTCA and Tail and Interacts With ODF1 and ODF2 in the Formation of the Male Germ Cell Cytoskeleton

**DOI:** 10.3389/fcell.2019.00151

**Published:** 2019-08-14

**Authors:** Constanza Tapia Contreras, Sigrid Hoyer-Fender

**Affiliations:** Johann-Friedrich-Blumenbach-Institute of Zoology and Anthropology – Developmental Biology, Göttingen Center for Molecular Biosciences (GZMB), Georg-August-University of Göttingen, Göttingen, Germany

**Keywords:** spermiogenesis, HTCA, tail, CCDC42, centrosome, ODF2, ODF1

## Abstract

Terminal differentiation of male germ cells into functional spermatozoa requires shaping and condensation of the nucleus as well as the formation of sperm-specific structures. A transient microtubular structure, the manchette, is mandatory for sperm head shaping and the development of the connecting piece and the sperm tail. The connecting piece or head-to-tail coupling apparatus (HTCA) mediates the tight linkage of sperm head and tail causing decapitation and infertility when faulty. Using mice as the experimental model, several proteins have already been identified affecting the linkage complex, manchette or tail formation when missing. However, our current knowledge is far too rudimentary to even draft an interacting protein network. Depletion of the major outer dense fiber protein 1 (ODF1) mainly caused decapitation and male infertility but validated binding partners collaborating in the formation of sperm-specific structures are largely unknown. Amongst all candidate proteins affecting the HTCA when missing, the structural protein CCDC42 attracted our attention. The coiled-coil domain containing 42 (CCDC42) is important for HTCA and sperm tail formation but is otherwise largely uncharacterized. We show here that CCDC42 is expressed in spermatids and localizes to the manchette, the connecting piece and the tail. Beyond that, we show that CCDC42 is not restricted to male germ cells but is also expressed in somatic cells in which it localizes to the centrosome. Although centrosomal and sperm tail location seems to be irrespective of ODF1 we asked whether both proteins may form an interacting network in the male germ cell. We additionally considered ODF2, a prevalent protein involved in the formation of spermatid-specific cytoskeletal structures, as a putative binding partner. Our data depict for the first time the subcellular location of CCDC42 in spermatids and deepen our knowledge about the composition of the spermatid/sperm-specific structures. The presence of CCDC42 in the centrosome of somatic cells together with the obvious restricted male-specific phenotype when missing strongly argues for a compensatory function by other still unknown proteins most likely of the same family.

## Introduction

The transformation of spermatids into terminally differentiated sperm is a key event in spermatogenesis. Meiosis II generates spermatids of spherical shape that are then gradually transformed by shaping and condensation of the nucleus and the formation of acrosome and sperm tail, finally resulting in the mature spermatozoon (Fawcett, 1975; Russell et al., 1990; Jan et al., 2012). There is circumstantial evidence indicating that the shaping of the nucleus and the assembly of the sperm tail is provoked by a transient microtubular structure, the manchette (Clermont et al., 1993; Kierszenbaum, 2002; Kierszenbaum et al., 2011). The manchette forms during the acrosomal phase of spermiogenesis and disassembles during the maturation phase (Clermont et al., 1993). In mice, it is first seen in step 8 spermatids and disassembles prior to the formation of the sperm mid-piece around steps 13–14 (O’Donnell and O’Bryan, 2014; Lehti and Sironen, 2016). The manchette consists of a perinuclear mantle of microtubules emanating from the perinuclear ring (Fawcett et al., 1971; Rattner and Brinkley, 1972; Dooher and Bennett, 1973; Rattner and Olson, 1973; Wolosewick and Bryan, 1977; Clermont et al., 1993). However, detection of plus-end tracking proteins as EB3 and CLIP-170 at the perinuclear ring together with the absence of the minus-end binding protein γ-tubulin strongly argues against the perinuclear ring as the microtubule nucleation site (Akhmanova et al., 2005; Kierszenbaum et al., 2011; Lehti and Sironen, 2016). Instead, supporting evidence indicates that the centriolar adjunct serves as a nucleator of manchette microtubules with their plus ends reaching toward the perinuclear ring (Fawcett and Phillips, 1969; Lehti and Sironen, 2016; Fishman et al., 2018). The manchette is linked to the nuclear membrane and this is essential for nuclear shaping. The presence of rod-like elements that link the manchette to the nuclear envelope has first been demonstrated by electron microscopy studies (Russell et al., 1991). Supportively, deletion of SUN4, a testis-specific nuclear membrane protein and component of the linker of nucleoskeleton and cytoskeleton complex (LINC) caused detachment of the manchette and consequently round-headed sperm (Calvi et al., 2015; Pasch et al., 2015; Yang et al., 2018a). The importance of the manchette for nuclear shaping and male fertility is furthermore exemplified by the seminal discovery of the genetic cause underlying the *azh* phenotype in mice. Male *azh* mice are infertile due to a malformed manchette, abnormal spermatozoon head morphology, tail abnormalities and decapitation all caused by a deletion in the Hook1 gene (Mendoza-Lujambio et al., 2002). (Review in: Chen et al., 2016). However, Hook1 is a microtubule-binding protein and most likely responsible for the cross-linking of the manchette microtubules, whereas SUN4 is expected to be an inner nuclear membrane protein. Thus, the true nature of the rod-like elements that link the manchette to the nucleus is still unknown.

The observation of sperm decapitation indicated that the manchette is involved in sperm head to tail coupling and/or development of the sperm tail. Consequently, it was suggested that molecules required for the developing basal body/connecting piece and the sperm tail were delivered via intra-manchette transport meaning that the manchette functions as a track in supporting the delivery of molecules (Kierszenbaum, 2001, 2002; Kierszenbaum et al., 2011). Contradictory, however, are the observations that the manchette is assembled when the axoneme is already developed and that the sperm tail develops irrespective of the detachment of the manchette in SUN4-deficient spermatids (Lehti and Sironen, 2017; Yang et al., 2018a).

The sperm tail develops from the basal body that itself is a derivative of the former centrosome. In spermatids, the daughter centriole of the centrosome is transformed into the proximal centriole, which acts as a seed for the formation of the connecting piece, and inserts into the nuclear indentation (Fawcett and Phillips, 1969). The perpendicular positioned mother centriole is transformed into the distal centriole, which acts as the basal body to initiate sperm tail development. Later on, the distal centriole disintegrates leaving the centriolar vault. The axoneme, the microtubule-based core structure, is the prolongation of the distal centriole that is surrounded by accessory structures as the nine prominent outer dense fibers (ODFs) and the fibrous sheath (FS) in the sperm tail. The ODFs are descending from the segmented columns formed at the proximal centriole of the head-to-tail coupling apparatus (HTCA). They accompany the microtubule doublets of the axoneme throughout the length of the tail whereas the FS is present only in the principal piece. The accessory fibers are important for stiffening the sperm tail thus supporting the elastic recoil of the sperm tail and protecting against shearing forces (Baltz et al., 1990; Lindemann, 1996). At the proximal region of the sperm tail, at the mid-piece, the mitochondrial sheath surrounds axoneme and ODFs.

The HTCA or connecting piece develops from the centrosome. It is an articular structure at the neck region mediating the tight connection between the sperm tail and the nucleus. Although the protein composition of the HTCA is far from being known, a couple of proteins have already been identified that are essential for the formation of the HTCA and/or the sperm tail. One protein essential for the tight connection of sperm head and tail is the outer dense fiber protein 1 (ODF1; also named HSPB10) (Burfeind and Hoyer-Fender, 1991; Schalles et al., 1998; Fontaine et al., 2003). Depletion of ODF1 caused sperm decapitation and male infertility in mice (Yang et al., 2012, 2014). A few interacting proteins have been identified, e.g., the outer dense fiber protein 2 (ODF2), which is a major protein of the sperm tail accessory fibers (Shao et al., 1997). Beyond that, validated ODF1 interacting proteins that are supposed to collaborate in the formation of sperm-specific structures are currently unknown. Proteins known to affect the HTCA or the sperm tail when missing are ideal candidates as putative interaction partners. We, therefore, focused on structural proteins with a reported effect on HTCA and sperm tail formation as putative interaction partners of ODF1. We asked here, whether the coiled-coil domain containing 42 (CCDC42) protein acts as a node in the ODF1 network. *Ccdc42* is specifically expressed in testis and brain and its deletion causes male sterility in mice with malformation of the HTCA and the sperm tail. Beyond that, no further phenotypes are evident (Pasek et al., 2016). CCDC42 (coiled-coil domain containing 42) belongs to the CFAP73 family and is a paralog of CFAP73. It contains the DUF4200, the domain of unknown function that is shared by a couple of coiled-coil domain proteins and cilia-and flagella-associated proteins as CFAP73. The phenotype of *Ccdc42*-deficient mice suggested a male germ cell-specific function, but its expression and sub-cellular location is so far unknown. We explored here putative interacting proteins of CCDC42 in male germ cells and analyzed its expression and sub-cellular location. We show that CCDC42 is recruited to the manchette and the sperm tail and is specifically enriched in the perinuclear ring of the manchette and the HTCA. Pull down and co-IP experiments both indicated binding to ODF1 and ODF2. Furthermore, CCDC42 localizes to the centrosome/basal body not only in male germ cells but also in somatic cells. Revision of *Ccdc42* expression by RT-PCR demonstrated wide-spread expression in somatic tissues. The co-localization of CCDC42 with microtubule-based structures as the manchette and the centrosome/HTCA suggests that CCDC42 is involved in their formation by generating a rigid scaffold. However, as no further phenotypes are evident when *Ccdc42* is missing its function in somatic cells most likely might be taken over by other members of the family.

## Materials and Methods

### Ethics Statement

All mouse experiments were reviewed and approved by the local ethic commission. License for animal experiments has been obtained by the Institute of Human Genetics and the Max-Planck-Institute for Experimental Medicine, Göttingen. The guidelines of the German Animal Welfare Act (German Ministry of Agriculture, Health and Economic Cooperation) were strictly followed in all aspects of mouse work.

### cDNA Synthesis and RT-PCR

Total RNA was prepared form adult mouse tissues as well as from NIH3T3 mouse fibroblasts using peqGOLD RNApure^TM^ (PeqLab, Erlangen, Germany) following the recommendations of the manufacturer. Total RNA was digested with Ambion^®^ TURBO DNA-free^TM^ DNase (Life Technologies) followed by cDNA synthesis using Maxima First Strand cDNA Synthesis (Thermo Fisher Scientific). RT-PCR for detection of transcribed sequences was performed using the following primer combinations: Ccdc42-Nterm_For (GTGGCACTGTCACTCACC) and C-terminal-Ccdc42-Rev (GGCTCACCAGGAACCTTCTC) generating the full-length product of 1093 bp, Ccdc42-For2 (GGAGACCGAGAATCCA GCC) and Ccdc42-Rev2 (CCGTTGGAATGCCTCCTTCT) for amplification of 305 bp of the 5′ region (exons 1 + 2), C-terminal-Ccdc42-For (GGAATCCACCCAAGTGTCCC) and C-terminal-Ccdc42-Rev (GGCTCACCAGGAACCTTCTC) generating a fragment of 203 bp of the conserved 3′ region (exons 6 + 7), Ccdc42-Exon5-For (GAAGAGATCCACGAGGTG) and C-terminal-Ccdc42-Rev for amplification of exons 5-7 (expected fragment size 535 bp), Gapdh-For (GTATGA CTCCACTCACGGCA) and Gapdh-Rev (GTCAGATCCACGA CGGACAC) generating a fragment of 594 bp.

### Plasmid Constructs

PCR amplification based on the Ensembl reference sequence NM_177779 by using the following primers: *Ccdc42-Nhe*I-*For* (5′-GGCTGTTAGGTAGCTAGCGCAAC CATGAGTTTGGG-3′) and *Ccdc42-Hin*dIII*-Rev* (5′-GTTA CTTCCTTAAGCTTGCCATCCGGACTTGCTGTbTG-3′), each primer containing restriction enzyme recognition sites. The full-length coding sequence of the *Ccdc42* isoform 203 was first cloned into pJET1.2/blunt (Thermo Fisher Scientific) followed by *Nhe*I/*Hin*dIII digestion and sub-cloning into pCR3.1-Cherry resulting in an in-frame fusion with the C-terminal Cherry-tag (pCR3.1, Invitrogen). The full-length coding region of *Odf1* was N-terminally fused to *ECFP* in *pECFP-C1* (Clontech Lab.). *Odf2* was C-terminally fused to *EGFP* in *pEGFP-N1* (Clontech Lab., #U55762) generating the full length construct *13.8NC-EGFP* (Donkor et al., 2004). Sequencing always revealed correct reading frames.

### Cell Culture and Immunocytochemistry

NIH3T3 (ATCC CRL-1658) or HEK-293 cells (ATCC CRL-1573) were maintained in Dulbecco’s Modified Eagle’s Medium (DMEM) supplemented with 10% (v/v) fetal bovine serum (FBS), 1000 U/ml penicillin, 1000 μg/ml streptomycin, and 20 mM L-Glutamine (all Gibco) at 37°C and 5% CO_2_. NIH3T3 cells were grown on coverslips in 6-well plates and transfected using EndoFectin^TM^ Max Transfection Reagent, following the recommendations of the manufacturer (GeneCopoeia). Twenty-four hours after transfection, cells were washed in phosphate-buffered saline (PBS) and fixed in methanol for 10 min at −20°C. Specimens were then permeabilized in 0.3% TritonX-100 in PBS for 10 min at room temperature and blocked for 1 h using blocking solution (PBS containing 1% BSA and 0.3% TritonX-100). Samples were incubated with primary antibodies toward CCDC42 (ARP52735_P050, antibodies-online ABIN2785068), Pericentrin (PRB432C, Covance), acetylated tubulin (6-11B-1; Sigma-Aldrich), gamma-tubulin (GTU-88, Sigma-Aldrich), ODF1 (ABIN4341345, antibodies-online), and GFP (raised in rabbit, self-made) at 37°C for 1 h. Secondary antibodies used are goat anti-mouse-IgG DyLight 488 (#35503, Thermo Fisher Scientific), and goat anti-rabbit MFP590 (#MFP-A1037, Mobitec). DNA was counterstained with DAPI. Images were taken by confocal microscopy (LSM 780, Zeiss) and processed using Adobe Photoshop 7.0.

### Immunocytology on Testicular Cell Suspensions

Fresh testes from laboratory mice of strain C57/Bl6, or frozen epididymides from wild-type mice or *Odf1*-ko mice (Yang et al., 2012) were minced in PBS, transferred onto superfrost slides, and fixed either in 2% or 3.7% paraformaldehyde in PBS for 20 min. Cells were permeabilized afterward in 0.3% Triton X-100 in PBS for 10 min, followed by blocking for 1 hr in blocking solution (PBS containing 1% BSA and 0.3% Triton X-100). The following antibodies were used for immunocytology: anti-α-tubulin (mouse monoclonal, DM1A, Calbiochem), anti-acetylated tubulin (6-11B-1, Sigma-Aldrich), anti-CCDC42 (ARP52735_P050; antibodies-online ABIN2785068), anti-ODF1 (antibodies-online, ABIN4341345), guinea pig anti-SUN4 (self-made, Manfred Alsheimer, Würzburg). Primary antibodies were detected using different combinations of secondary antibodies as goat anti-mouse-IgG Alexa Fluor 555 IgG (H + L) (A21422, Molecular Probes) and goat anti-rabbit-IgG DyLight 488 (#35553, Thermo Fisher Scientific), goat anti-mouse-IgG DyLight 488 (#35503, Thermo Fisher Scientific) and goat anti-rabbit-MFP590 (#MFP-A1037, Mobitec), goat anti-mouse-IgG DyLight 488 (#35503, Thermo Fisher Scientific) and goat anti-rabbit-IgG (H + L) Alexa Fluor R 555 (F[ab]2 fragment; #A21430, Life Technologies), and goat anti-guinea pig-IgG Cy3 (Dianova #106-166-003). DNA was counterstained with DAPI (4′, 6-Diamidino-2-phenylindole; Sigma D-9542), and the acrosome was decorated with FITC-labeled peanut lectin (PL-FITC). Images were taken by confocal microscopy (LSM 510, Zeiss) and processed using Adobe Photoshop 7.0. In some pictures, the fluorescent colors are replaced by pseudo-colors.

### Co-immunoprecipitation

HEK-293 cells were transfected using EndoFectin^TM^ Max Transfection Reagent (GeneCopoeia), and 24 h post-transfection harvested by trypsinization followed by two rinses in PBS. The cell pellet was resuspended in 1 ml lysis buffer (150 mM NaCl, 1% Nonidet P40, 0.5% sodium deoxycholate, 0.1% SDS, 50 mM Tris-HCl, pH 7.6, containing protease inhibitor cocktail (Halt Protease Inhibitor Cocktail 100x, Thermo Fisher Scientific, #78438). Subsequently, cell lysates were passed 10x through a syringe with a 21-gauge needle, followed by 3-times sonication for 45 s each. The soluble fraction was obtained by centrifugation at 15,000 × *g* for 15 min at 4°C. The supernatant was split into three parts and incubated with different antibodies for co-IP, either with rabbit anti-GFP (self-made), rat anti–RedFP (5F8, Chromotek), or rabbit anti-GAL4 (DBD) (sc-577, Santa Cruz Biotechnology). Protein G agarose beads (Thermo Fisher Scientific) were washed three times with lysis buffer and the antibody/supernatant mixture was then added. Samples were incubated overnight at 4°C on a rotating wheel. Afterward, beads were washed four times with lysis buffer, and bound proteins obtained by suspension of beads in SDS sample buffer and heating at 95°C for 10 min. Proteins were fractionated on SDS-PAGE and transferred onto nitrocellulose membrane (Amersham Hybond-ECL, GE Healthcare) (Laemmli, 1970; Towbin et al., 1979). The membrane was incubated in blocking solution [5% dry milk in TBST (10 mM Tris-HCl, pH 7.6, 150 mM NaCl 0.05% Tween 20)] for 1 h. Membranes were incubated overnight at 4°C with primary antibodies either rabbit anti-GFP (self-made), mouse anti-GFP (MAB3580, Chemicon), or rat anti-RedFP (5F8, Chromotek) in blocking solution. Following washing of membranes in TBST, they were incubated with horseradish peroxidase-conjugated secondary antibodies either anti-rabbit IgG, anti-mouse IgG, or anti-rat IgG (Jackson ImmunoResearch, WestGrove, PA, United States or Sigma Biosciences, St Louis), respectively. Chemiluminescence detection was performed using ClarityMax Western ECL Substrate (Bio-Rad, #1705062) and images captured with Chemdoc (Bio-Rad).

### Pull Down Assay

Preparation of bacterially expressed and refolded His-tagged ODF2-fusion protein (6xHis-13.8NC) was essentially as described in Yang et al. (2018b). Ni-NTA agarose (Qiagen GmbH, Hilden) was washed in wash buffer (50 mM NaPi, 500 mM NaCl, 30 mM imidazole, pH 7.6, containing protease inhibitors (Halt Protease Inhibitor Cocktail 100x, Thermo Fisher Scientific, #78438) and 0.2 mM PMSF). For *in vitro* interaction, 6xHis-ODF2 proteins were added to the washed Ni-NTA agarose and incubated for 1 h at 4°C in constant agitation. NIH3T3 cell lysate containing CCDC42-Cherry proteins was split into halves and one half added to the resin followed by incubation for 2 h at 4°C and constant agitation. The second half was used for the negative control. The beads were washed four times in wash buffer, followed by a final overnight washing step. To elute bond proteins, Ni-NTA agarose was incubated for 10 min at 4°C in elution buffer (50 mM NaPi, 500 mM NaCl, 500 mM imidazole, 0.2 mM PMSF). Eluates were boiled in SDS-sample buffer and analyzed by Western-blotting using rabbit anti-ODF2 antibodies (ESAP 15572, ABIN2430582, antibodies-online) and rat anti-RedFP antibodies (5F8, Chromotek). As negative control, the resin was incubated with one half of the NIH3T3 cell lysate containing CCDC42-Cherry proteins but without 6xHis-ODF2 proteins and processed as described.

## Results

### CCDC42 Localizes to Manchette, Perinuclear Ring, Connecting Piece and Sperm Tail

The coiled-coil domain containing protein of 42 kDa (CCDC42) belongs to the DUF4200 family of proteins containing the domain of unknown function 4200. Important paralogs of CCDC42 are CFAP73, CCDC38, and CFAP100, also known as CCDC37/MIA1. CCDC42 orthologs are present in most organisms from vertebrates to choanoflagellates (Ruan et al., 2008; Guindon et al., 2010). Despite their widespread occurrence, information is currently scarce. *Ccdc42* is specifically expressed in testis and brain but deletion of *Ccdc42* seems to affect exclusively male germ cells resulting in infertility (Pasek et al., 2016). *Ccdc42*-deficient spermatids developed malformed HTCA and sperm tail that are functionally insufficient. However, beyond that no further information about CCDC42 is available. We first studied the subcellular localization of CCDC42 during spermatogenesis in the mouse using a commercially available antibody. The antibody was first validated by immunocytology and Western blotting using the *Ccdc42*-expression construct (validation results are online at antibodies-online and are shown in Figure 9^1^).

**FIGURE 1:**
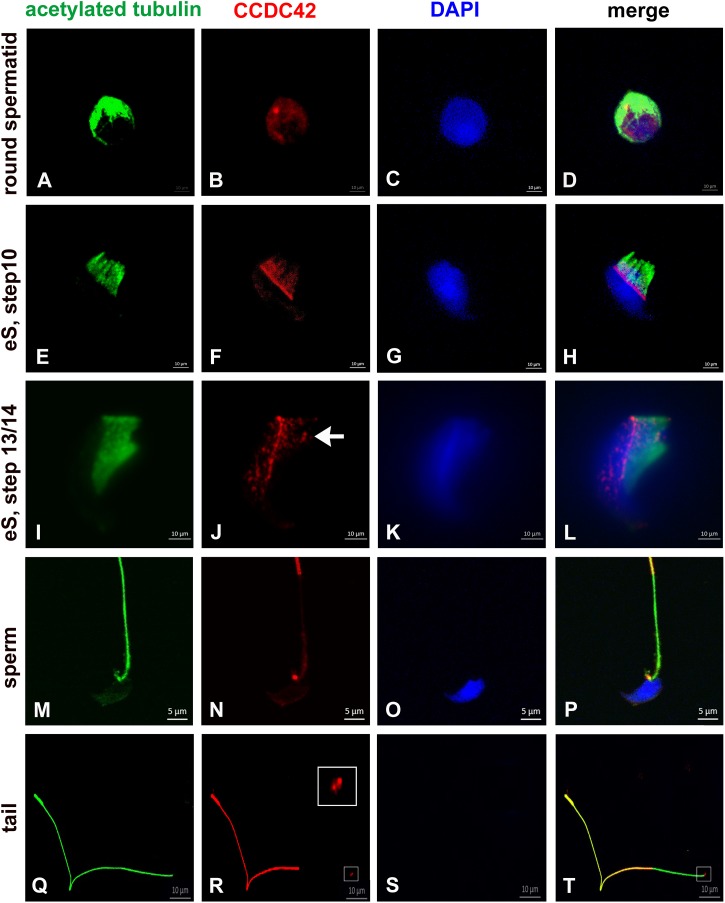
CCDC42 localizes in the manchette, the perinuclear ring and the connecting piece during spermiogenesis. Suspension preparations of adult mouse testis were incubated with antibodies against acetylated tubulin (green) and CCDC42 (red). Weak staining for CCDC42 was observed in the cytoplasm in round spermatids **(A–D)**. In elongating spermatids (eS) CCDC42 decorated the manchette and more strongly the perinuclear ring **(E–L)**. Additionally, the HTCA or connecting piece harbored CCDC42 as well (**I–L**, arrow in **J**). CCDC42 also located to the connecting piece in sperm **(M–P)** and to the tail with highest expression in the principal piece **(M–T)**. In detached tails, the anterior region, which corresponds to the attachment site to the nucleus, stained for CCDC42 (**Q–T**; framed in **R** and **S** and inset in **R** showing the enlarged region). Secondary antibodies used are anti-mouse IgG-Dylight488 and anti-rabbit IgG-MFP590 **(A–H,M–P)** or anti-rabbit IgG-Dylight488 and anti-mouse IgG-Alexa Fluor R 555 **(I–L,Q–T)**. Nuclear staining with DAPI (blue). Bars are of 10 μm except for **M–P** in which 5 μm scales are used.

Weak expression of CCDC42 was first observed in the cytoplasm of round spermatids (Figures 1A–D). In elongating spermatids CCDC42 co-localized with the manchette microtubules decorated by acetylated tubulin and more strongly with the perinuclear ring which marks the anterior border of the manchette (Figures 1E–L) Additionally, CCDC42 localized to the connecting piece detectable as two adjacent spots at the posterior end of the nucleus (Figures 1I–L, arrow in J). In sperm, CCDC42, again, was found at the connecting piece region and located to the sperm tail. CCDC42 strongly decorated the principal piece but only weakly the middle piece whereas acetylated tubulin marked the whole tail (Figures 1M–P). Labeling of detached sperm tails confirmed prevalent localization of CCDC42 in the principal piece whereas acetylated tubulin marked the whole tail. However, the anterior region of detached sperm tails, which corresponds to the former attachment site of the tail to the head, showed presence of CCDC42 visible by two adjacent spots (Figures 1Q–T; framed in R, S and enlarged inset in R). A stronger staining of the principal piece of the sperm tail than of the mid-piece might either reflect an unequal distribution of CCDC42 along the sperm tail or is caused by different accessibilities of the antibodies due to the presence of the mitochondrial sheath in the mid-piece.

**FIGURE 2:**
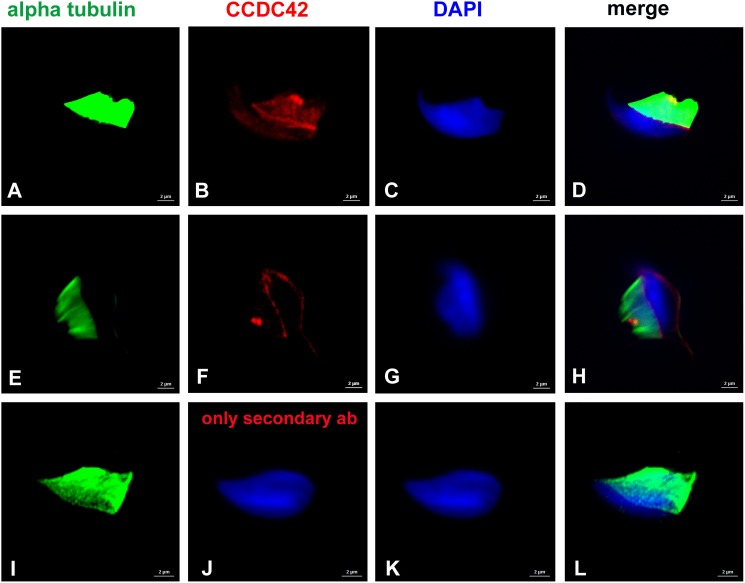
CCDC42 locates to manchette, perinuclear ring and connecting piece. Suspension preparations of adult mouse testis were incubated with antibodies against α-tubulin (**A,E,I**; green) and CCDC42 (**B,F**; red) and subsequently detected with the secondary antibodies anti-mouse IgG Dylight488 and anti-rabbit IgG MFP590 **(A–D,I–L)** or anti-mouse IgG-Dylight488 and anti-rabbit IgG-Alexa Fluor R 555 **(E–H)**. In the control, the antibody against CCDC42 was omitted **(I–L)**. Nuclear counterstain with DAPI (blue). Bars: 2 μm.

Decoration of the manchette, the perinuclear ring, and the connecting piece was also demonstrated by using anti-α-tubulin antibody staining in conjunction with anti-CCDC42, both antibodies subsequently detected by varying secondary antibodies (Figure 2). The control, with anti-α-tubulin antibody incubation but omitting anti-CCDC42 antibody, followed by both secondary antibodies subsequently, revealed only α-tubulin staining thus supporting specificity of anti-CCDC42 staining (Figures 2I–L).

**FIGURE 3:**
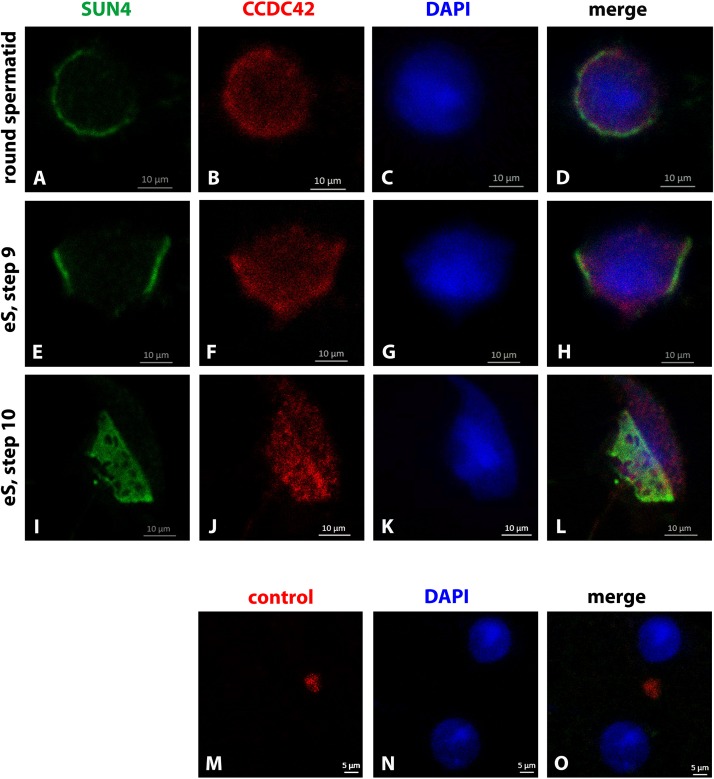
Partial overlap of CCDC42 and SUN4 location. Mouse testicular cell suspensions were incubated with antibodies against SUN4 (green) and CCDC42 (red). In the round and early elongating spermatids (eS, step 9) a partial overlap between SUN4 and CCDC42 positive domains was observed **(A–H)**. The fuzziness of the CCDC42 positive domain, however, suggests a cytoplasmic rather than a nuclear envelope location as for SUN4. In elongating spermatids of step 10, the concentration of CCDC42 at the perinuclear ring is obvious once more **(I–L)**. In the control, without primary antibody, no CCDC42 was observed **(M–O)**. Nuclear counterstain with DAPI (blue). Bars are of 10 μm except for **M–O** in which scale bars are of 5 μm.

When concurrently stained for the nuclear envelope protein SUN4, the weak CCDC42 staining of the cytoplasm in round and early elongating spermatids was confirmed (Figures 3A–H). The SUN4 positive domain partially overlapped with the CCDC42 positive region as this is the region where the manchette develops. In early elongating spermatids, the SUN4 and CCDC42 localization domains seemingly overlapped corresponding most likely to the region where the manchette has formed and to which SUN4 locates (I-L) (Yang et al., 2018a). Again, CCDC42 more strongly decorated the perinuclear ring (I–L). The weak cytoplasmic staining for CCDC42 in the round spermatid seemed to be beyond background staining as demonstrated by the control experiment (Figures 3M–O).

### CCDC42 Interacts With ODF1 and ODF2

**FIGURE 4:**
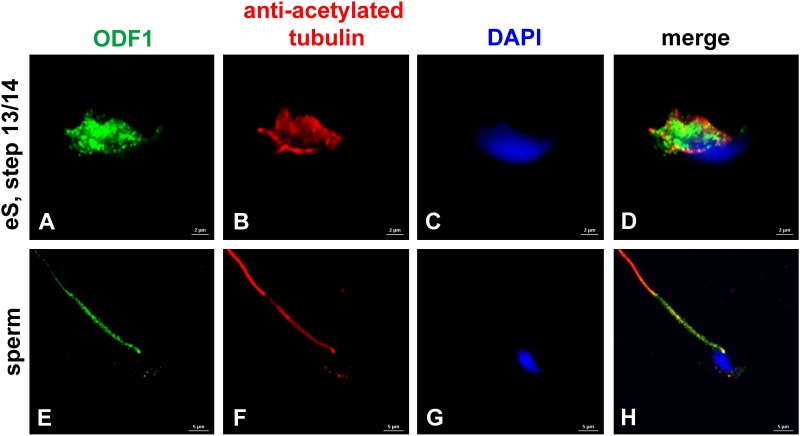
ODF1 locates to the manchette in elongating spermatids and to the sperm tail. Incubation of mouse testicular cells with antibodies against ODF1 (green) and acetylated tubulin (red). ODF1 decorates the manchette in elongating spermatids **(A–D)** and the sperm tail **(E–H)**. Nuclear counterstain with DAPI (blue). Bars: 2 μm **(A–D)**, 5 μm **(E–H)**.

The small heat shock protein ODF1/HSPB10 is a main protein component of the sperm tail ODFs. Beyond that, it locates to the connecting piece and is essential for the tight connection between head and tail (Schalles et al., 1998; Yang et al., 2012, 2014). Immunocytological inspection confirmed sperm tail location of ODF1 and, additionally, showed expression in the manchette of elongating spermatids (Figure 4). Location of ODF1 thus resembled that of CCDC42 raising the question whether both proteins also physically interact.

**FIGURE 5:**
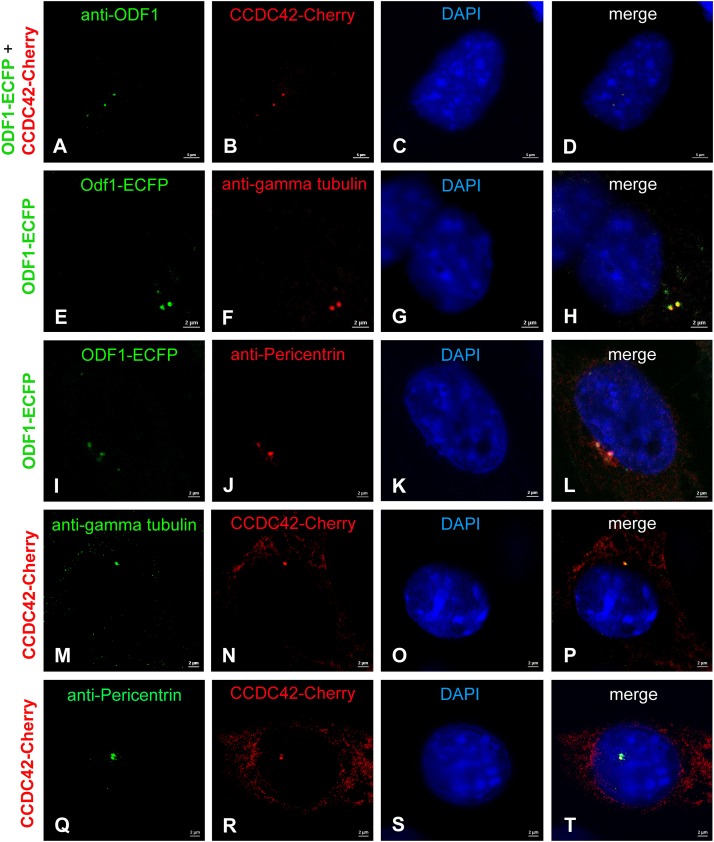
Co-localization of CCDC42-Cherry and ODF1-ECFP in NIH3T3 cells. NIH3T3 cells were transfected with expression plasmids encoding either CCDC42-Cherry or ODF1-ECFP as marked on the left side. Proteins were detected either by their fluorescent tag (CCDC42-Cherry or ODF1-ECFP) or by immunostaining (anti-ODF1, anti-gamma tubulin, or anti-Pericentrin). **(A–D)** CCDC42-Cherry co-localizes with ODF1-ECFP when ectopically expressed. Ectopically expressed ODF1-ECFP localizes to the centrosome as verified by immunostaining using the centrosomal marker proteins γ-tubulin **(E–H)** or Pericentrin **(I–L)**. Immunostaining for γ-tubulin **(M–P)** or Pericentrin **(Q–T)** demonstrates centrosomal location of CCDC42-Cherry as well. Nuclear counterstain with DAPI (blue). Bars are of 5 μm **(A–D)** or 2 μm **(E–T)**.

We first investigated whether the location of ODF1 and CCDC42 is interdependent when ectopically expressed in NIH3T3 mouse fibroblasts. Cells were transfected with expression plasmids encoding either CCDC42 fused to Cherry (CCDC42-Cherry) or ODF1 fused to ECFP (ODF1-ECFP) and the proteins detected either by their fluorescent tags or by immunostaining (Figure 5). Ectopic expression of CCDC42-Cherry revealed bright staining of one or two dots close to the nucleus that overlap with ODF1-ECFP expression (Figures 5A–D). Since twin-dots are a typical signature of the centrosome, we verified centrosomal location of ODF1-ECFP (Figures 5E–L) as well as of CCDC42-Cherry (Figures 5M–T) using immunostaining for the centrosomal marker proteins γ-tubulin (Figures 5E–H,M–P) or Pericentrin (Figures 5I–L,Q–T) (Doxsey et al., 1994). Our results show that CCDC42-Cherry co-localizes with ODF1-ECFP, when both proteins are ectopically expressed in NIH3T3 cells, notably at the centrosome. However, recruitment of CCDC42-Cherry to the centrosome is independent of ODF1 due to the fact that ODF1 is not at all expressed in somatic cells and CCDC42 locates to the centrosome despite absence of ODF1-ECFP (Figures 5M–T) (Yang et al., 2012).

**FIGURE 6:**
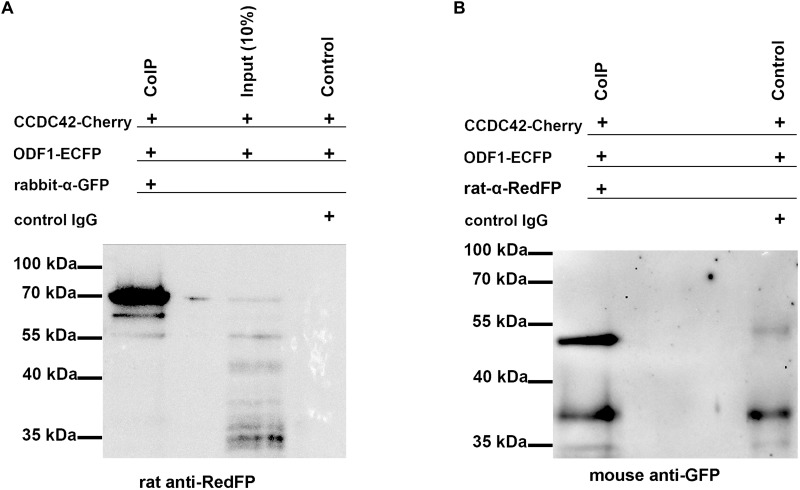
Co-precipitation of CCDC42 and ODF1. Cells were transfected with the expression plasmids encoding CCDC42-Cherry or ODF1-ECFP. Proteins were immunoprecipitated from cell lysates using either anti-GFP (raised in rabbit, rabbit anti-GFP; **A**) to capture ODF1-ECFP or anti-RedFP (raised in rat, rat anti-RedFP; **B**) to capture CCDC42-Cherry. The co-purified protein interacting with the captured protein was detected by immunoblotting using either anti-RedFP **(A)** or anti-GFP **(B)** as first antibodies followed by detection of first antibodies using the secondary antibodies anti-rat IgG (for detection of CCDC42-Cherry) or anti-mouse IgGs (for detection of ODF1-ECFP). ODF1-ECFP, captured by rabbit anti-GFP antibody, co-precipitated CCDC42-Cherry with an expected molecular mass of ∼70 kDa. **(A)** When CCDC42-Cherry was captured using the rat anti-RedFP-antibody **(B)**, ODF1-ECFP was co-purified as well **(B)**. Immunoblotting using the first antibody against GFP raised in mouse **(B)**, followed by the secondary anti-mouse IgG antibody, detected the expected ODF1-ECFP protein of ∼54 kDa but only weakly reacted with the control IgGs, which were raised in rabbit (control) and which show a slightly higher molecular mass.

**FIGURE 7:**
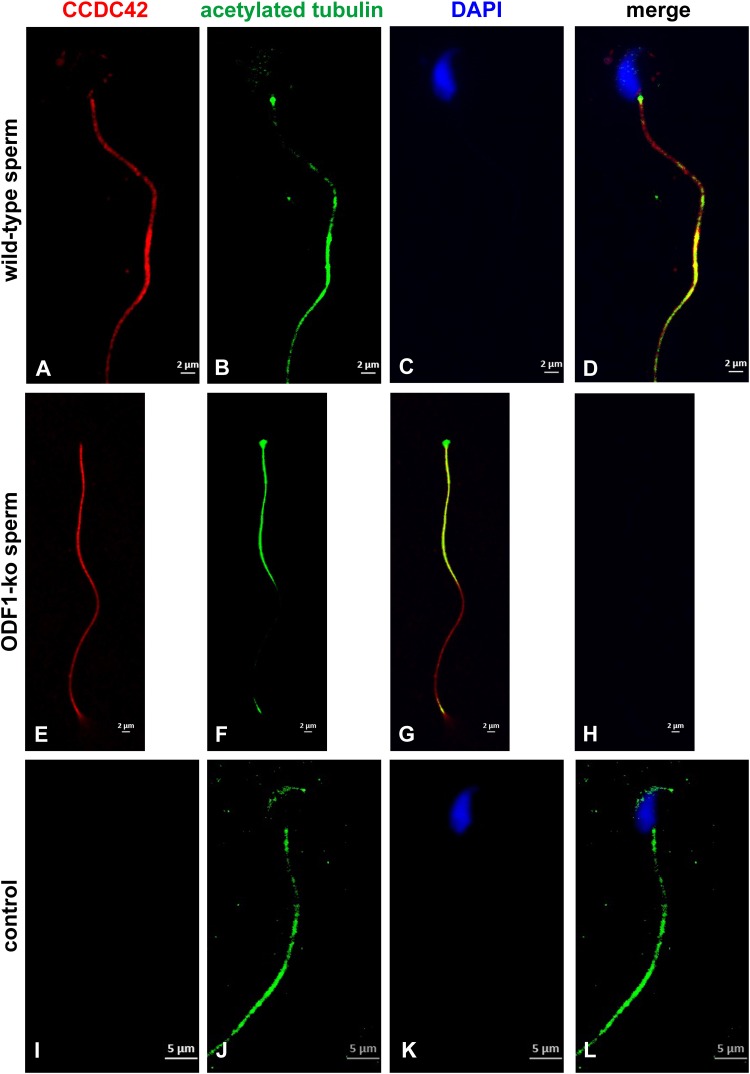
ODF1 is dispensable for recruitment of CCDC42 to the sperm tail. Epididymal sperm of wild-type mice **(A–D)** and *Odf1*-ko mice **(E–H)** were decorated for acetylated tubulin (green) and CCDC42 (red). Nuclear staining with DAPI. Loss of ODF1 caused head detachment. The sperm head is, therefore, missing in *Odf1*-ko sperm **(E–H)**. No red staining was evident when anti-CCDC42 antibody incubation was omitted (**I–L**, control). **A–H**: 2 μm bars, **I–L**: 5 μm bars.

Nevertheless, the physical interaction between CCDC42-Cherry and ODF1-ECFP was proven by co-immunoprecipitation. Both proteins were ectopically expressed in cultured cells by transient transfection of expression plasmids, and one of either protein immunoprecipitated out of the cell lysate. The immunoprecipitate was analyzed by immunoblotting detecting the protein that co-precipitated with the fished protein (Figure 6). Co-immunoprecipitation of CCDC42-Cherry and ODF1-ECFP was verified in either direction, capturing either CCDC42-Cherry or ODF1-ECFP. Our data thus indicate physical interaction between CCDC42 and ODF1. However, ODF1 is not only dispensable for recruitment of CCDC42 to the centrosome but also to the sperm tail. Immunocytology on epididymal sperm of *Odf1*-ko mice showed decoration for CCDC42 similar as in wild-type sperm (Figure 7, wild-type sperm in A-D, *Odf1*-ko sperm in E-H). Acetylated tubulin staining identified the sperm tail. As ODF1 is essential for the anchorage of the sperm head to the tail causing head detachment when missing, the sperm head is absent in ODF1-ko sperm (Figures 7E–H) (Yang et al., 2012). In the control staining, when omitting anti-CCDC42 incubation but subsequent incubation with both secondary antibodies, the sperm tail is clearly visible by acetylated tubulin decoration but missed any red staining (Figures 7I–L).

**FIGURE 8:**
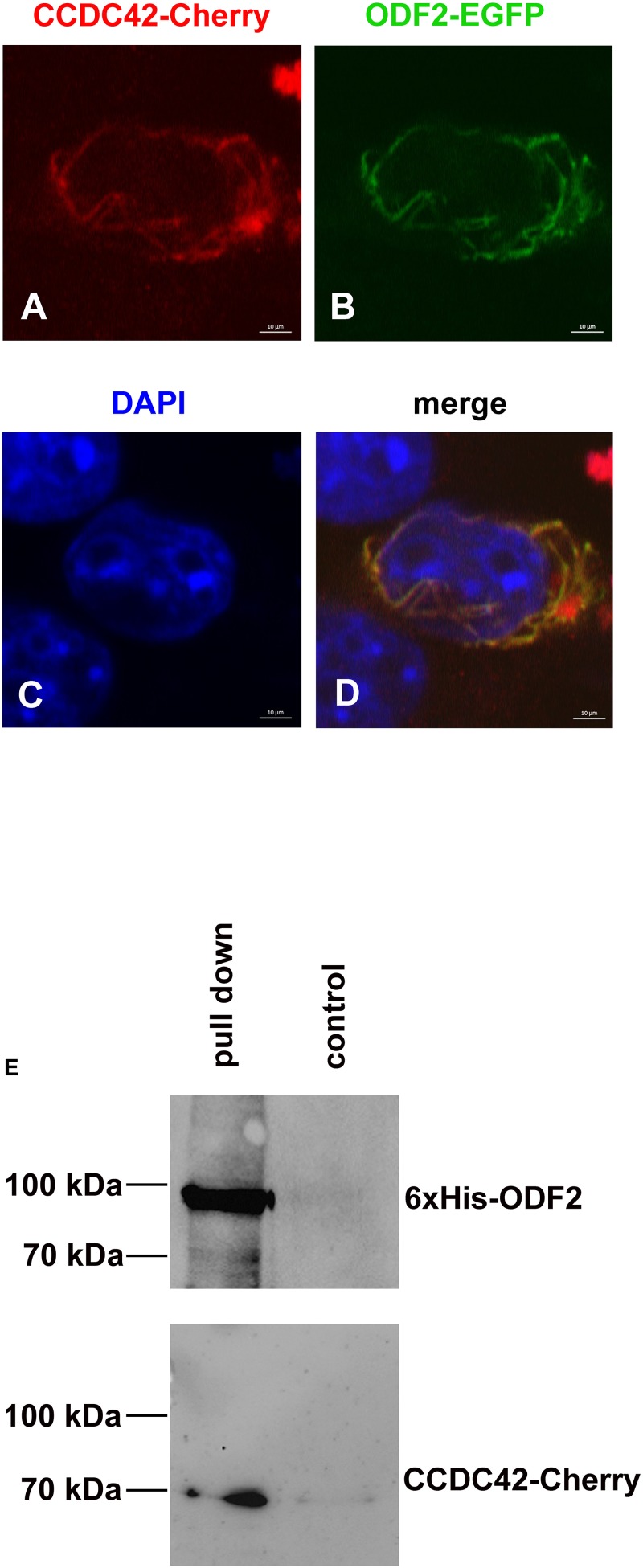
CCDC42 interacts with ODF2. **(A–D)** Co-transfection of expression plasmids encoding ODF2-EGFP (green) or CCDC42-Cherry (red) in NIH3T3 cells and detection by their fluorescent tags. Fusion proteins co-localize in the cytoplasm. Nuclear staining with DAPI (blue). Bars of 10 μm. **(E)** *In vitro* interaction of His-tagged ODF2 (6xHis-ODF2/6xHis-13.8NC) and Cherry-tagged CCDC42. Co-purification of bacterially expressed 6xHis-ODF2 and CCDC42-Cherry, ectopically expressed in NIH3T3 cells, by affinity purification of 6xHis-ODF2 using Ni-NTA agarose (pull down). Low binding of CCDC42-Cherry to the agarose beads in the absence of 6xHis-ODF2 (control). Proteins were eluted from the beads, and analyzed by immunoblotting using antibodies against ODF2 (6xHis-ODF2) or RedFP (CCDC42-Cherry).

Another important protein of the sperm tail and the connecting piece is ODF2. ODF2, furthermore, is an essential component of the centrosome and the basal body in somatic cells (Brohmann et al., 1997; Schalles et al., 1998; Nakagawa et al., 2001; Hüber et al., 2008). We, therefore, asked whether ODF2 is another binding partner of CCDC42. Co-transfection assays of expression plasmids in NIH3T3 cells revealed similar location of ODF2-EGFP and CCDC42-Cherry. ODF2 fused to EGFP (13.8NC-EGFP) often generated fibrous structures to which CCDC42-Cherry proteins localize (Figures 8A–D). Additionally, a physical interaction between CCDC42-Cherry and ODF2 was proven by pull-down assays (Figure 8E). Bacterially expressed and refolded 6xHis-tagged ODF2 was affinity purified using Ni-NTA agarose in the presence of CCDC42-Cherry, ectopically expressed in cell culture. Thereafter, eluates were immunoblotted for detection of the target protein 6xHis-ODF2 and its putative binding partner CCDC42-Cherry. CCDC42-Cherry co-purified with 6xHis-ODF2 (Figure 8E, pull down), whereas almost no binding of CCDC42-Cherry to the beads was observed in the absence of 6xHis-ODF2 (Figure 8E control). Our data, therefore, indicate ODF2 as another binding partner of CCDC42.

### CCDC42 Is a Centrosomal Protein in Somatic Cells

**FIGURE 9:**
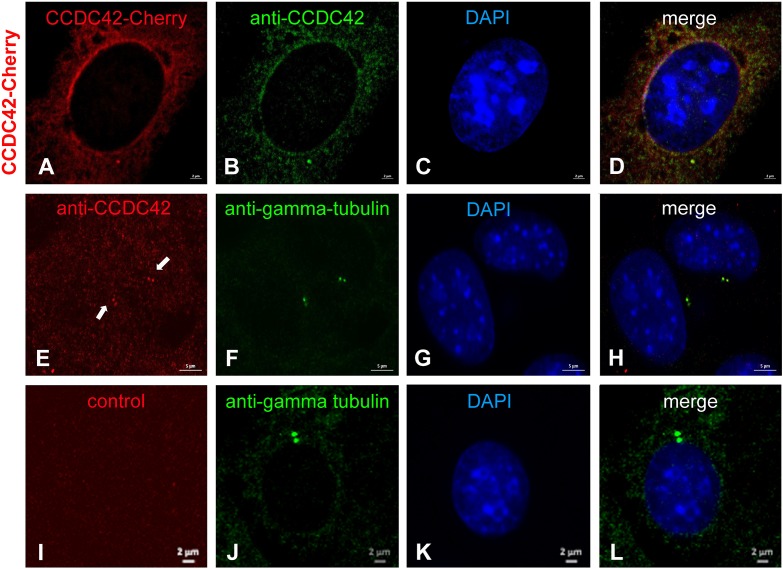
CCDC42 is a centrosomal component in NIH3T3 cells. **(A–D)** NIH3T3 cells were transfected with *Ccdc42-Cherry* expression plasmid (as indicated on the left side by CCDC42-Cherry) and CCDC42 detected by its fluorescent tag (CCDC42-Cherry, red) and immuno-decoration (anti-CCDC42, green) to validate the antibody. **(E–H)** Endogenous expression of CCDC42 in NIH3T3 cells was detected immunocytologically (anti-CCDC42, red) and co-located with the centrosomal marker protein γ-tubulin (anti-gamma-tubulin, green). **(I–L)** Omitting anti-CCDC42 antibody incubation showed no red decoration of the centrosome, which was otherwise detected by anti-γ-tubulin staining (anti-gamma-tubulin, green), demonstrating anti-CCDC42 antibody specificity. Nuclear counterstain with DAPI (blue). Bars are of 2 μm **(A–D,I–L)** or of 5 μm **(E–H)**.

By ectopic expression of CCDC42-Cherry in NIH3T3 cells we have observed a predominant centrosomal location (Figure 5). This prompted us to investigate whether CCDC42 is endogenously expressed in somatic cells being a novel component of the centrosome. We first transfected cells with the CCDC42-Cherry expression plasmid for validation of the anti-CCDC42 antibody (Figures 9A–D). The antibody specifically decorated only transfected cells and co-localized with the fusion protein CCDC42-Cherry thus demonstrating its validity (Figures 9A–D). We next incubated untransfected NIH3T3 cells with the anti-CCDC42 antibody. Immunostaining of the endogenous CCDC42 decorated a twin-spot near the nucleus that additionally stained for the centrosomal marker γ-tubulin (Figures 9E–H). The centrosome was exclusively decorated by γ-tubulin staining but did not show a red fluorescence when omitting anti-CCDC42 antibody incubation (Figures 9I–L, control). Immunocytological data thus indicate expression of CCDC42 in somatic cells, which is contradictory to its reported restricted expression pattern.

### Expression of Ccdc42 Isoforms

**FIGURE 10:**
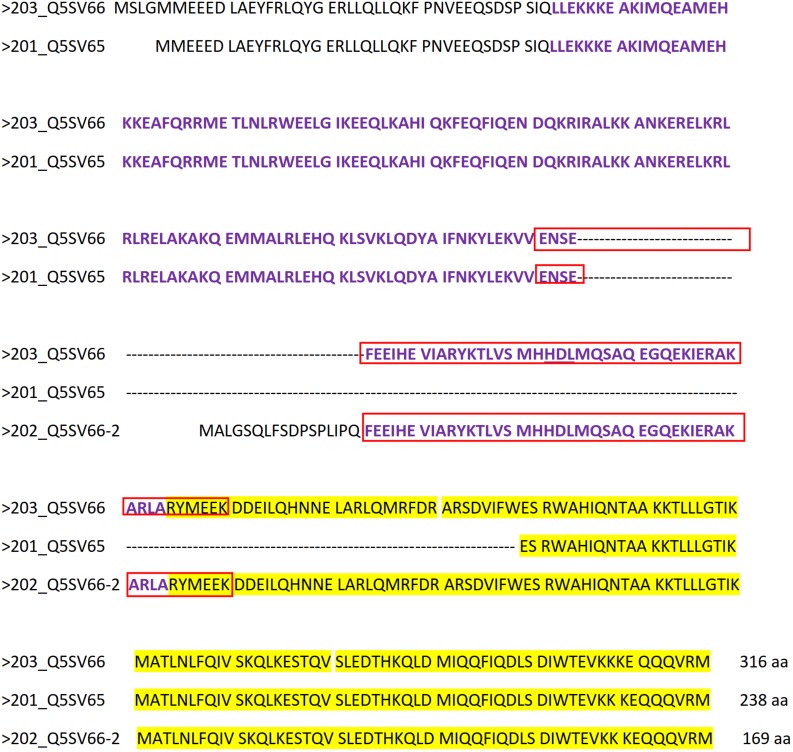
Amino acid alignment of reported CCDC42 isoforms. The longest isoform 203 (Q5SV66) consists of 316 aa. In isoform 201 (Q5SV65) the sequence encoded by exon 5 is missing resulting in a putative protein of 238 aa. Isoform 202 (Q5SV66-2) has a postulated length of 169 aa since the N-terminal end encoded by exons 1-4 is completely missing. Instead, translation of the protein starts at the 3′ end of intron 4 encoding sequence MALGSQLFSDPSPLIPQ. The domain of unknown function 4200 (DUF4200), comprising aa 44-161 at the N-terminal half of isoform 203, is depicted in violet. The coiled-coil region is highlighted in yellow. The epitope, to which antibody ABIN2785068 is directed, is framed in red.

According to Pasek et al. (2016), *Ccdc42* is expressed in testis and brain. In mouse testes, weak expression was first observed at 10-days of age that raised in 15-days old testis and maintained into adulthood. Testicular expression thus corresponds with the onset of meiosis around day 10 and its increase roughly coincides with the progression of spermatid differentiation during spermiogenesis (Nebel et al., 1961). However, three putative CCDC42 isoforms have been reported in mice (UniProtKB – Q5SV66) produced by alternative splicing (Figure 10). The longest isoform 203 (Q5SV66) consists of 316 amino acids (aa). In isoform 201 (Q5SV65) the sequence encoded by exon 5 is missing resulting in a putative protein of 238 aa. Isoform 202 (Q5SV66-2) has a postulated length of 169 aa since the N-terminal end encoded by exons 1-4 is completely missing. Instead, translation of the protein starts at the 3′ end of intron 4 encoding sequence MALGSQLFSDPSPLIPQ upstream of exon 5 encoded sequences. According to Interpro, the domain of unknown function 4200 (DUF4200) comprises aa 44-161 at the N-terminal half of isoform 203 and is therefore present in both, isoform 203 as well as in isoform 201, albeit shortened in the latter, but is largely missing in isoform 202 (Figure 10, depicted in violet). The coiled-coil region at the C-terminal end is present in all three isoforms (according to SMART; in Figure 10 highlighted in yellow). The antibody ABIN2785068, used for immunocytology, detects the epitope of 50 aa in the DUF4200, which is present in isoforms 203 and 202 but not in 201 (Figure 10, enframed in red).

**FIGURE 11:**
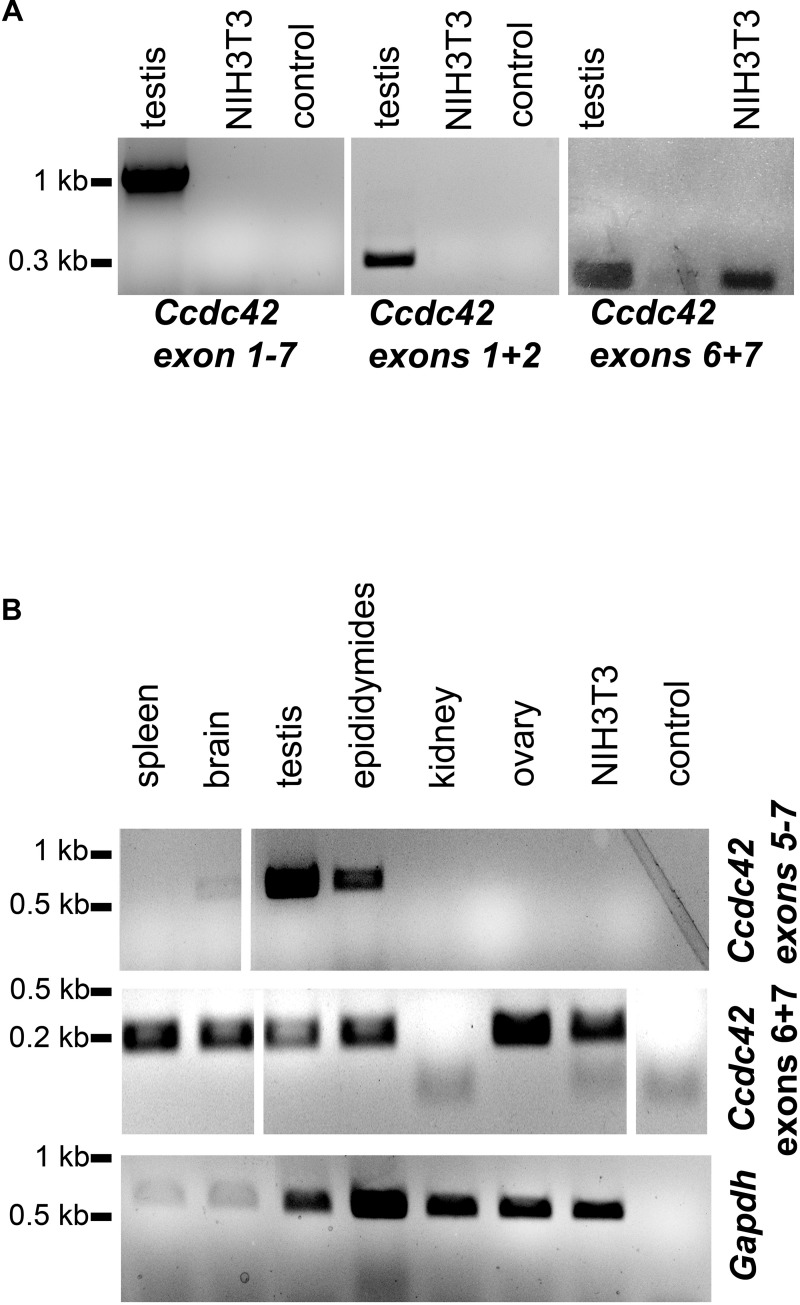
Expression of *Ccdc42* is not restricted to testis. **(A)** RT-PCR on cDNA generated from adult mouse testis as well as from NIH3T3 mouse fibroblasts using different primer combinations for the amplification of the full-length cDNA (exons 1-7), exons 1 + 2, and exons 6 + 7. The full-length cDNA of 1093 bp corresponding to isoform 203, as well as the 5′ exons 1 + 2 of 305 bp were exclusively amplified from testis cDNA but not from NIH3T3 cells whereas the 3′ exons 6 + 7 of 203 bp were also amplified from NIH3T3 cells. **(B)** Nested PCR for the detection of *Ccdc42* expression. First RT-PCR on cDNA synthesized from mouse tissues to amplify exons 5-7 revealed a fragment of 535 bp in brain, testis, and epididymides. A nested PCR on the first RT-PCR products was performed with primers amplifying exons 6 + 7. In all tissues investigated, with the exception of kidney, the expected fragment of 203 bp was found. Amplification of *Gapdh* as control. Probes were loaded side by side on one gel but uninformative lanes were skipped from the picture as indicated by white lines.

Expression of *Ccdc42* was reported to be restricted to testis. In somatic tissues, with the only exception being the brain, *Ccdc42* seems to be not expressed. We confirmed testicular expression by RT-PCR using different primer combinations (Figure 11). The full-length product of isoform *Ccdc42-203* was expected to be of 1093 bp, which was confirmed by RT-PCR (Figure 11A, exons 1–7). However, for the putative isoform *Ccdc42*-*201*, a length of 729 bp was expected since exon 5 was skipped but we could not amplify the expected fragment (Figure 11A). Amplification of 305 bp of the 5′ region (exons 1 + 2) again confirmed testicular expression of *Ccdc42* (Figure 11A, exons 1 + 2). When amplifying 203 bp of the conserved 3′ region, *Ccdc42* expression was also demonstrated in NIH3T3 mouse fibroblasts (Figure 11A, exons 6 + 7).

Our results thus indicate that expression of *Ccdc42* isoforms is not restricted to testis. This prompted us to revise *Ccdc42* expression in other tissues (Figure 11B). We performed a nested PCR in order to detect even low expression levels. The first RT-PCR was performed to amplify exons 5–7 encoding part of the DUF domain with the epitope detected by the antibody, and the conserved coiled-coil region. We found strong expression in testis and epididymides and weak expression in the brain. Sequencing of the RT-PCR products generated form testis and epididymides confirmed *Ccdc42*. *Gapdh* amplification, albeit demonstrating successful cDNA synthesis in all tissues, also indicated low amounts of cDNA in spleen and brain, which most likely accounts for the weak RT-PCR band found in brain cDNA. When performing a nested PCR on the first PCR products by amplifying exons 6 + 7 a fragment of the expected size was found in all tissues, with the exception of kidney. The RT-PCR fragments generated in ovary and NIH3T3 cDNA were sequenced confirming *Ccdc42* amplification. Our results, therefore, indicate that *Ccdc42* expression is not restricted to testis. However, since the full-length product and the 5′ region (exons 1 + 2) were found only in testis but not in NIH3T3 cells it is most likely that in somatic cells another isoform than the full-length CCDC42 isoform 203 is predominantly expressed, and that this isoform was detected by immunocytology in the centrosome.

## Discussion

The HTCA is a complex structure present in the neck region of the sperm interconnecting the head and the tail. It develops from the centrosome that itself is composed of a pair of centrioles and associated components. During spermiogenesis, the proximal centriole inserts into a nuclear indentation, known as the implantation fossa, opposed to the acrosomal cap, and the linkage complex and the longitudinal columns of the connecting piece are formed (Fawcett and Phillips, 1969). The sperm tail starts outgrowing from the distal centriole of the former centriole that is now the basal body. In order to transmit only one centriole during fertilization, sperm of most vertebrates have disintegrated the distal centriole leaving only the proximal centriole. In contrast to centriole reduction in most vertebrates, in mice and other rodents, both distal and proximal centrioles degenerate during spermiogenesis leaving the centriolar vaults (Schatten, 1994; Hoyer-Fender, 2011). The current dogma of centrosome reduction in sperm was recently revisited by investigating the centrosomal protein inventory in human sperm. These data showed that the distal centriole is remodeled into an atypical centriole surrounded by a pericentriolar matrix instead of being completely vanished (Fishman et al., 2018). It is, therefore, feasible to view the neck structure as a specialized form of the pericentriolar matrix, and the centrioles as nucleation site for both the sperm tail and the manchette MTs. Few proteins are currently known that affect the head to tail linkage when missing, in between ODF1 and CCDC42 (Yang et al., 2012; Pasek et al., 2016). ODF1 is located in the sperm tail ODFs and in the connecting piece (Schalles et al., 1998). It interacts with ODF2, the major outer dense fiber protein, but no further interacting proteins have been confirmed (Shao et al., 1997). To figure out the interrelationship of HTCA proteins, and how they function in the formation of the HTCA, ODF1 interacting proteins are of utmost importance.

The coiled-coil domain containing 42 protein CCDC42 is highly conserved in evolution with orthologs existing in most organisms from vertebrates to choanoflagellates (Ruan et al., 2008; Guindon et al., 2010). Additionally, important paralogs of CCDC42 (also named CCDC42A) exist as CFAP73 (also named CCDC42B and MIA2), CCDC38 and CFAP100 (also named CCDC37 and MIA1). They altogether constitute the CFAP73 protein family. These proteins are in essential coiled-coil domain proteins and share the domain of unknown function DUF4200. Coiled-coil domain containing proteins are often involved in ciliary motility (Inaba and Mizuno, 2016; Zur Lage et al., 2019). In *C. reinhardtii* the gene product of the CCDC42 homolog MIA2 is a dynein regulator and necessary for ciliary motility (Yamamoto et al., 2013). Further coiled-coil domain proteins as CCDC39 and CCDC40 are essential for ciliary motility by assembly of the dynein regulatory complex (Becker-Heck et al., 2011; Merveille et al., 2011; Blanchon et al., 2012). *Ccdc42*-ko mice are phenotypically normal but males are sterile. Sterility of *Ccdc42*-deficient male mice is most likely caused by the malformation of the HTCA and the sperm tail resulting in functional insufficiency (Pasek et al., 2016). These data suggest that CCDC42 has an important function in male germ cells but is otherwise dispensable (Pasek et al., 2016). Since CCDC42-deficiency affected exclusively the male germ cell, CCDC42 seems not to be involved in ciliary motility because otherwise, a more generalized phenotype has to expected. CCDC42-deficient spermatids are characterized by a multiplicity of the HTCA, defective nuclear shaping despite presence of a manchette, dislocation of the HTCA from its implantation site and a loss of flagellar outgrowth from the HTCA (Pasek et al., 2016).

We have demonstrated by immunocytology that CCDC42 co-localizes with ODF1- and ODF2-fusion proteins when ectopically expressed, and with structures comprising these proteins endogenously. Furthermore, co-precipitation by pull-down and co-immunoprecipitation experiments indicated binding of CCDC42 to ODF1 as well as to ODF2. However, recruitment of CCDC42 to the sperm tail and the centrosome in somatic cells does not require ODF1. CCDC42 consists in essential of coiled-coil domains, which is also the main feature of ODF2. Coiled-coil domains are important oligomerization domains mediating homodimerization as well as heterodimerization (Mason and Arndt, 2004). It is, therefore, most likely that ODF2 and CCDC42 interact by means of their coiled-coil domains. This mutual interaction might contribute to the stabilization of important cytoskeletal structures potentially mediated by ODF1. Although the true molecular function of ODF1 is not known, it belongs to the small heat shock protein family and might, therefore, act as a chaperone in protein folding (Fontaine et al., 2003). The protein complex consisting of the core proteins ODF2/ODF1/CCDC42 may thus build the rigid scaffold essential for the formation of the connecting piece and the sperm tail. When missing any one of these proteins the rigid scaffold is damaged causing failure of the linkage complex and the sperm tail. Whether CCDC42 interacts with any of those proteins that have a reported function in HTCA formation, as Centrin 1, Centrobin, Spata6, or Azi1/Cep131 awaits further investigation (Liska et al., 2009; Avashti et al., 2013; Hall et al., 2013; Yuan et al., 2015). However, since CCDC42-deficient sperm often show two instead of one basal body inserted into the nuclear membrane, centriole duplication, as well as correct attachment of the centrioles to the implantation fossa seem to be affected (Pasek et al., 2016). As similar phenotypes have been observed concerning mutations in Centrobin, Centrin 1, and Azi/Cep131 a functional interaction with CCDC42 is likely (Liska et al., 2009; Avashti et al., 2013; Hall et al., 2013). Our observation that CCDC42 is expressed in the manchette and in particular in the perinuclear ring illuminates its involvement in the acrosome-acroplaxome complex formation and nuclear shaping that are both affected when CCDC42 is missing. Since loss of CCDC42 did not prevent manchette formation, it is most likely involved in stabilizing the manchette or is a passenger protein transported via the manchette. Its accumulation in the perinuclear ring, however, points toward a stabilizing function and its involvement in the attachment of the manchette to the nuclear membrane.

Our data show that CCDC42 expression is not restricted to testis and brain but instead is found also in somatic tissues. We detected the endogenous protein in the centrosome of somatic NIH3T3 cells and hence identified CCDC42 as a novel component of the centrosome and the sperm tail not found before by large scale proteomics screens (Amaral et al., 2013; centrosome database Centrosome:DB). However, albeit RT-PCR experiments confirmed expression of *Ccdc42* in somatic tissues, the full-length sequence could only by amplified from testis cDNA. It is therefore probable that the full-length isoform 203 is restricted to testis or more specifically to male germ cells whereas another isoform is expressed in somatic tissues. We could not verify expression of isoform 202, which starts with translated sequences encoded by intron 4 since a primer that binds to these 5′ sequences has amplified an unrelated almost unknown sequence (C6H1orf158). Furthermore, we got no indications by RT-PCR of isoform 201, which was expected to be encoded by a smaller cDNA due to skipping of exon 5. Our data additionally show that CCDC42 is a component of the centrosome in somatic cells and most likely functions in scaffolding the centrosome via interaction with ODF2/Cenexin. However, since the only obvious phenotype of CCDC42-deficient mice is male infertility, CCDC42 is either dispensable for the somatic centrosome or its function has been taken over by other members of the CFAP73 family.

## Data Availability

The datasets generated for this study are available on request to the corresponding author.

## Author Contributions

CTC did the experiments and prepared the figures. SH-F was the project leader and wrote the manuscript. Both authors read and approved the final manuscript.

## Conflict of Interest Statement

The authors declare that the research was conducted in the absence of any commercial or financial relationships that could be construed as a potential conflict of interest.

## Footnotes

1 https://www.antibodies-online.com
